# Treatment of corn with lactic acid or hydrochloric acid modulates the rumen and plasma metabolic profiles as well as inflammatory responses in beef steers

**DOI:** 10.1186/s12917-018-1734-3

**Published:** 2018-12-18

**Authors:** You Yang, Guozhong Dong, Zhi Wang, Junhui Liu, Jingbo Chen, Zhu Zhang

**Affiliations:** College of Animal Science and Technology, Southwest University, Chongqing, 400716 People’s Republic of China

**Keywords:** Metabolomics, Beef steers, Lactic acid, Hydrochloric acid, Corn

## Abstract

**Background:**

High-grain diets that meet the energy requirements of high-producing ruminants are associated with a high risk of rumen disorders. Mild acid treatment with lactic acid (LA) has been used to modify the degradable characteristics of grains to improve the negative effects of high-grain diets. However, the related studies mainly focused on dairy cows and explored the effects on rumen fermentation, production performance, ruminal pH and so forth. And up to date, no studies have reported the hydrochloric acid (HA) treatment of grains for ruminant animals. Therefore, based on metabolomics analysis, the aim of this study was to evaluate the effects of treatment of corn by steeping in 1% LA or 1% HA for 48 h on the rumen and plasma metabolic profiles in beef steers fed a high corn (48.76%) diet with a 60:40 ratio of concentrate to roughage. The inflammatory responses of beef cattle fed LA- and HA-treated corn were also investigated.

**Results:**

Based on ultra-high-performance liquid tandem chromatography-quadrupole time-of-flight mass spectrometry (UHPLC-QTOF/MS) metabolomics and multivariate analyses, this study showed that steeping corn in 1% LA or 1% HA modulated the metabolic profiles of the rumen. Feeding beef steers corn steeped in 1% LA or 1% HA was associated with lower relative abundance of carbohydrate metabolites, amino acid metabolites, xanthine, uracil and DL-lactate in the rumen; with higher ruminal pH; with lower concentrations of acetate, iso-butyrate and iso-valerate; and with a tendency for lower ruminal lipopolysaccharide (LPS) concentrations. Moreover, the data showed lower concentrations of plasma C-reactive protein, serum amyloid A, haptoglobin, interleukin (IL)-1β and IL-8 in beef steers fed 1% LA- or HA-treated corn. The 1% LA treatment decreased the concentrations of plasma LPS, LPS-binding protein and tumour necrosis factor-alpha and the relative abundance of L-phenylalanine, DL-3-phenyllactic acid and tyramine in plasma. The 1% HA treatment decreased the relative abundance of urea in plasma and increased the relative abundance of all amino acids in the plasma.

**Conclusions:**

These findings indicated that LA or HA treatment of corn modulated the degradation characteristics of starch, which contributed to improving the rumen and plasma metabolic profiles and to decreasing inflammatory responses in beef steers fed a high-concentrate diet.

**Electronic supplementary material:**

The online version of this article (10.1186/s12917-018-1734-3) contains supplementary material, which is available to authorized users.

## Background

For ruminants, the efficiency of dietary energy utilization is greater for starch derived from cereals than for cellulose derived from forages [1]. Today’s intensive feedlot management systems encourage large amounts of cereal grains in the diets of steers to enhance fattening. As a result, excessive amounts of carbohydrates cause volatile fatty acid (VFA) accumulation, ruminal pH depression, and ruminal microbiota dysbiosis, which could increase the possibility of developing subacute ruminal acidosis (SARA) [2, 3]. When SARA occurs, a large amount of bacterial lipopolysaccharide (LPS) is released in the rumen and the large intestine [4, 5]. When rumen LPS is translocated into the blood circulation, an inflammatory response is activated [5, 6]. Entry of LPS into blood circulation can also result in metabolic disorders [7]. The negative effects of high-grain diets will decrease the long-term production of beef cattle.

Thus, strategies to prevent high-grain diets from causing SARA are highly desirable. Over the years, substantial research efforts have been made to modulate the rumen degradability of grain by physical and chemical processing, aiming to improve grain feed efficiency and its health benefits [8–10]. Reynolds considered that the majority of the supplemental energy arising from postruminal starch digestion is used with high efficiency to support body adipose and protein retention [1]. Deckardt reviewed the most important achievements in the chemical processing to date and considered that mild acids are a promising method [11]. Among these organic acids, lactic acid (LA) has received more attention [12–14], and the results demonstrated that LA processing modulated rumen fermentation patterns, increased rumen pH and productivity, and improved cow health. However, this method might require more research to confirm its mechanism of lowering the amount of rumen fermentable starch from the metabolomics point of view. In addition, no previous investigations on the effects of steeping grain in LA on beef cattle have been conducted.

Hydrochloric acid (HA) is an inorganic acid and the component of the gastric acid secreted by the abomasum. Moreover, HA has a higher acidity than LA and the p*K*_a_ value of HA and LA is − 7.0 and 3.9, respectively [15]. Therefore, we hypothesized that HA treatment should be as effective as LA treatment for slowing the starch degradation rate of grain in the rumen.

Metabolomics is an emerging field of “omics” science that uses high-throughput approaches coupled with multivariate analysis to extract comprehensive metabolic information and measured metabolic phenotypes in mammals, plants, and microbes [16–18]. Therefore, based on the fact that corn is the main grain feedstuff in many countries, the aim of this study was to evaluate the effects of feeding corn steeped in LA or HA on the metabolic profiles of the rumen and plasma in beef steers by Ultra-high-performance liquid tandem chromatography-quadrupole time-of-flight mass spectrometry (UHPLC-QTOF-MS). In addition, the inflammatory responses of beef cattle fed LA- and HA-treated corn was investigated.

## Methods

### Animals, diet and experimental design

Eighteen Charolais × Luxi hybrid steers of the Southwest University (Chongqing, China) experimental station were selected for this experiment. Steers born in the same season of calving and weaned and grown under the same conditions of management and feeding were used in the experiment. At the beginning of the experiment, the animals were 13 months of age and their initial body weight was 368 ± 52 kg with a similar body condition score. These steers were randomly assigned to 1 of 3 diets according to the completely randomized design. The 3 diets were identical, with the only difference being the treatment of corn: 48.76% (dry matter basis) corn grain in the diet was steeped for 48 h in an equal quantity (i.e., in a 1:1 ratio, wt/vol) of tap water (CON), 1% lactic acid (LA) or 1% hydrochloric acid (HA) before being mixed with other ingredients of the total mixed ration (TMR). The LA (L(+)-lactic acid, 85%, wt/wt) used in this study was purchased from Chengdu Cologne Chemical Co., Ltd. (Chengdu, China), and HA (hydrochloric acid, 37%, wt/wt) was purchased from Chongqing Chuandong Chemical Co., Ltd. (Chongqing, China). The steers were offered a TMR with a concentrate to roughage ratio of 60:40 (Table 1).

**Table 1:** Ingredients and nutrient composition of the experimental diets

Items	Diet^a^		
	LA	HA	CON
Ingredients, % of DM			
Chinese wildrye	11.50	11.50	11.50
Sorghum distiller’s grains	28.50	28.50	28.50
Corn grain (water-treated)	–	–	48.76
Corn grain (LA-treated)	48.76	–	–
Corn grain (HA-treated)	–	48.76	–
Rapeseed meal	8.61	8.61	8.61
Salt	0.30	0.30	0.30
Limestone	2.05	2.05	2.05
Premix^b^	0.28	0.28	0.28
Nutrients, % of DM			
Crude protein	13.5	13.8	13.6
Ether extract	4.2	4.2	4.2
Neutral detergent fibre	34.5	34.6	34.4
Acid detergent fibre	14.3	13.7	14.4
Crude ash	5.1	4.8	4.8
Calcium	0.35	0.36	0.38
Total phosphorus	0.31	0.29	0.31
Nonfiber carbohydrate^c^	42.7	42.6	43.0
Starch in corn grain	63.4	66.0	71.8

^a^LA is the treatment diet based on corn grain steeped for 48 h in an equal quantity of tap water containing 1% lactic acid (wt/vol), HA is the treatment diet based on corn grain steeped for 48 h in an equal quantity of tap water containing 1% hydrochloric acid (wt/vol), and CON is the control diet containing corn grain steeped for 48 h in an equal quantity of tap water

^b^Formulated to provide the following (per kg diet): Fe (as ferrous sulfate), 50 mg; Cu (as copper sulfate), 10 mg; Mn (as manganese sulfate), 20 mg; Co (as cobaltous sulfate), 0.1 mg; Zn (as zinc sulfate), 30 mg; I (as potassium iodate), 0.5 mg; Se (as sodium selenite), 0.1 mg; vitamin A, 2240 IU; vitamin D_3_, 500 IU; vitamin E, 40 IU; riboflavin, 6.2 mg; nicotinic acid, 22 mg; D-pantothenic acid, 22 mg; vitamin B_12_, 0.02 mg; biotin, 0.15 mg; and choline, 0.92 mg

^c^Nonfiber carbohydrate (%) = 100 - (% neutral detergent fibre + % crude protein + % ether extract + % crude ash)

The experimental period was 32 d, with the first 7 d used for an adaptation period. The steers were housed in individual tie stalls and had free access to feed and water. Diets were fed two times daily at 0630 and 1730 h to allow 5–10% orts. During the trial, the amount of feed offered and orts was recorded each day, and dry matter intake (DMI) for each cattle was calculated based on the dry matter (DM) contents of the diets. The DMI data were then used to calculate average daily dry matter intake (ADMI). After the experiment, all beef cattle were kept by stockmen of the university experimental station.

### Sample collection

Blood samples (10 mL per steer) were obtained from the jugular vein on day 20 before the morning feeding and were collected into sterile and pyrogen-free tubes containing sodium heparin. Blood samples were stored on ice and then centrifuged at 4 °C and 3000×g for 15 min to separate plasma. The plasma was transferred to 2 mL pyrogen-free tubes and immediately stored at − 80 °C until further analysis.

Four beef steers in each treatment were selected randomly for collection of the rumen fluid at the end of the experimental period. Three hours after the morning feeding, the rumen fluid was collected by an oral stomach tube equipped with a strainer and a 150 mL pyrogen-free syringe as described by Shen et al. [19]. When the length of the tube inserted into the rumen was approximately 200 cm, the rumen fluid was obtained with a 150 mL pyrogen-free syringe. The initial 50 mL rumen fluid was discarded to avoid saliva contamination. Subsequently, approximately 150 mL of rumen fluid was strained through four layers of sterile cheesecloth and filtrate was divided into two parts: one part was immediately used to determine the pH value by the portable pH meter (Rex PHS-3E, Shanghai INESA Scientific Instrument Co., Ltd., Shanghai, China), while the other part was transferred into sterile and pyrogen-free centrifuge tubes (approximately 50 mL per cattle) and centrifuged at 4 °C and 10,000×g for 30 min, and the supernatant was stored in 2 mL pyrogen-free tubes at − 80 °C until further analysis.

### Analyses of volatile fatty acids

VFA concentrations in the rumen fluid were determined using gas chromatograph (GC-2010, Shimadzu, Japan) with SH-RTX-WAX capillary columns (30 m × 0.25 μm × 0.25 mm, Shimadzu, Japan). One mL ruminal fluid supernatant was added to 0.2 mL metaphosphoric acid solution (25%), and vortexed for 30 s. After kept at 5 °C for 5 h, the samples were centrifuged at 14500×g for 15 min at 4 °C. The supernatant was transferred into a 1-mL glass vial for gas chromatograph analysis. One μL aliquot of the analyte was injected in split mode (50:1). The samples were run at a programmed temperature gradient (100 °C initial temperature for 1 min, with a 5 °C rise per min until 190 °C, and 190 °C for 15 min). N_2_ gas was used as the carrier gas, and the column flow rate was 1 mL min^− 1^. The temperature of the injector and detector was 230 °C and 240 °C, respectively.

### Analyses of lipopolysaccharide, cytokines, and acute phase proteins

The LPS concentrations in the rumen fluid and plasma; the concentrations of acute phase proteins in plasma, including C-reactive protein (CRP), serum amyloid A (SAA), haptoglobin (Hp), and LPS-binding protein (LBP); and plasma cytokines concentrations, including interleukin (IL)-1β, IL-6, IL-8 and tumour necrosis factor-alpha (TNF-α), were determined using a commercially available bovine ELISA kit. A bovine LPS ELISA kit was purchased from Shanghai Preferred Biotechnology Co., Ltd. (Shanghai, China). Bovine SAA, CRP, Hp and LBP ELISA kits were purchased from Nanjing Jianchen Saihao Biotechnology Co., Ltd. (Nanjing, China). Bovine IL-1β, IL-6, IL-8 and TNF-α ELISA kits were purchased from Shanghai Jinma Biotechnology Co., Ltd. (Shanghai, China). According to the manufacturer’s instructions, all samples were tested in duplicate, and the optical density values were read at 450 nm on an automatic microplate reader (EIX808IU; Biotek, Winooski, VT).

### Metabolomics analysis

All samples of rumen fluid and plasma were thawed at 4 °C on ice. Then, 100 μL of sample was taken, placed in an Eppendorf tube, and then extracted with 300 μL of methanol and 20 μL of internal standard substances for 30 s vortexes. Then, the extracts were ultrasound treated for 10 min (incubated in ice water) and incubated for 1 h at − 20 °C to precipitate proteins. Then, the samples were centrifuged at 12000×g for 15 min at 4 °C. In total, 200 μL of supernatant was transferred to LC-MS vials and stored at − 80 °C until the UHPLC-QTOF/MS analysis [20].

LC-MS/MS analyses were carried out on a UHPLC system (1290, Agilent Technologies) with a UPLC BEH amide column (2.1 mm × 100 mm × 1.7 μm, Waters) coupled to a Triple TOF 6600 (Q-TOF, AB Sciex). Elution was performed with a mobile phase of A (25 mM ammonium acetate and 25 mM ammonium hydroxide in water, pH = 9.75) and B (acetonitrile) under the following gradient program: 0 min, 5% A and 95% B; 7 min, 35% A and 65% B; 9 min, 60% A and 40% B; 9.1 min, 5% A and 95% B; and 12 min, 5% A and 95% B. The flow rate was 0.5 mL/min, and the injection volume was 2 μL for positive ion mode (POS) and 3 μL for negative ion mode (NEG).

A Triple TOF mass spectrometer was used to acquire MS/MS spectra on an information-dependent basis (IDA) during an LC/MS experiment. In this mode, the acquisition software (Analyst TF 1.7, AB Sciex) continuously evaluated the full scan survey MS data as it collected and triggered the acquisition of MS/MS spectra depending on preselected criteria. In each cycle, 12 precursor ions whose intensity was greater than 100 were chosen for fragmentation at collision energy (CE) of 30 V (15 MS/MS events with a product ion accumulation time of 50 msec each). The electron spray ionization (ESI) source conditions were set as follows: ion source gas 1 as 60 Psi, ion source gas 2 as 60 Psi, curtain gas as 35 Psi, source temperature as 650 °C, ion spray voltage floating (ISVF) 5000 V or − 4000 V in positive or negative mode, respectively [21].

### Data processing and statistical analyses

Data of ADMI; the rumen pH values and the concentrations of VFA; the LPS concentrations in the plasma and rumen fluid; plasma concentrations of SAA, CRP, Hp and LBP; and plasma concentrations of IL-1β, IL-6, IL-8 and TNF-α were analysed using ANOVA of SAS (SAS Institute, 2000). Data are presented as the means ± standard deviation. Means among treatments were compared using Duncan’s multiple range test. Statistical significance was defined as *P* ≤ 0.05.

MS raw data (.d) files were converted to the mzXML format by ProteoWizard (http://proteowizard.sourceforge.net/downloads.shtml) and processed by R package XCMS (https://xcmsonline.scripps.edu/landing_page.php?pgcontent=mainPage). The preprocessing results generated a data matrix that consisted of the retention time (RT), mass to charge ratio (m/z) values, and peak intensity [22]. The R package CAMERA was used for peak annotation after XCMS data processing. An in-house MS2 database was applied for metabolite identification [23].

The SIMCA software package (V14.1, Umea, Sweden) was used for pattern recognition multivariate analysis, including principal component analysis (PCA) and orthogonal partial least-squares discriminant analysis (OPLS-DA). Based on the OPLS-DA results, metabolites were plotted according to their importance in separating the dietary groups, and each metabolite received a value called variable importance for the projection (VIP). If VIP exceeded 1.0, the metabolite was first selected as the changed variable. These variables were then assessed using one-way ANOVA analysis. If *P* ≤ 0.05, the variables were defined as the significantly differential metabolites among the 3 groups [24].

## Results

### Feed intake

The ADMI of the CON, LA and HA groups was 6.82 ± 0.71, 6.33 ± 0.85 and 6.50 ± 0.69 kg, respectively. There was no significant difference in ADMI among the three diets (*P =* 0.53).

### The ruminal pH and the concentrations of volatile fatty acids in the rumen

Data for the ruminal pH and VFA contents are shown in Table 2. Compared with the ruminal pH value of the CON group, those of the LA and HA groups were higher (*P* < 0.01). Both LA-treated and HA-treated groups had lower acetate, iso-butyrate and iso-valerate (*P* < 0.05), while propionate, butyrate and valerate were not affected (*P* > 0.05).

**Table 2:** The ruminal pH and volatile fatty acids concentrations in the rumen of beef steers fed different diets

Items	Diet^1^			*P*-value
	LA	HA	CON	
Ruminal pH	6.74 ± 0.16^A^	6.73 ± 0.17^A^	6.22 ± 0.07^B^	0.0008
Acetate (mmol/L)	35.68 ± 2.79^A^	37.58 ± 5.15^A^	52.89 ± 9.03^B^	0.0066
Propionate (mmol/L)	26.08 ± 2.83	27.92 ± 9.41	28.46 ± 6.79	0.8787
Butyrate (mmol/L)	7.38 ± 0.68	7.11 ± 1.94	9.27 ± 1.70	0.1532
Iso-butyrate (mmol/L)	0.64 ± 0.10^a^	0.59 ± 0.03^a^	0.85 ± 0.20^b^	0.0408
Iso-valerate (mmol/L)	1.40 ± 0.17^A^	1.74 ± 0.51^A^	2.75 ± 0.47^B^	0.0033
Valerate (mmol/L)	0.61 ± 0.14	0.66 ± 0.20	0.91 ± 0.28	0.1627

^A-B^Means within a row differ (*P* < 0.01)

^a-b^Means within a row differ (*P* < 0.05)

^1^LA is the treatment diet based on corn grain steeped for 48 h in an equal quantity of tap water containing 1% lactic acid (wt/vol), HA is the treatment diet based ong corn grain steeped for 48 h in an equal quantity of tap water containing 1% hydrochloric acid (wt/vol), and CON is the control diet containing corn grain steeped for 48 h in an equal quantity of tap water

### The lipopolysaccharide concentrations in the rumen fluid and plasma

The effects of LA and HA on LPS concentrations in the rumen fluid and plasma are shown in Table 3. The lowest LPS concentrations in plasma were observed in beef cattle fed the LA-treated corn (*P* < 0.05).

**Table 3:** The lipopolysaccharide (LPS) concentrations in rumen fluid and plasma of beef steers fed different diets

Items	Diet^1^			*P*-value
	LA	HA	CON	
LPS (rumen, EU/mL)^2^	21,284.45± 1253.38	23,326.95± 2945.41	25,392.15± 1553.99	0.0572
LPS (plasma, EU/mL)	0.39 ± 0.06^a^	0.48 ± 0.08^b^	0.49 ± 0.06^b^	0.0380

^A-B^Means within a row differ (*P* < 0.01)

^a-b^Means within a row differ (*P* < 0.05)

^1^LA is the treatment diet based on corn grain steeped for 48 h in an equal quantity of tap water containing 1% lactic acid (wt/vol), HA is the treatment diet based on corn grain steeped for 48 h in an equal quantity of tap water containing 1% hydrochloric acid (wt/vol), and CON is the control diet containing corn grain steeped for 48 h in an equal quantity of tap water

^2^*EU* endotoxin unit

### Plasma acute phase proteins concentrations

As shown in Table 4, compared with the CON group, the LA and HA groups had significantly decreased CRP, SAA and Hp concentrations in plasma (*P* < 0.05). Moreover, the LA treatment had decreased LBP concentrations in plasma relative to the other groups (*P* < 0.05).

**Table 4:** The acute phase proteins concentrations in plasma of beef steers fed different diets

Items	Diet^1^			*P*-value
	LA	HA	CON	
CRP^2^ (μg/mL)	22.95 ± 4.57^A^	15.04 ± 5.30^A^	36.03 ± 9.35^B^	0.0003
SAA (μg/mL)	80.01 ± 13.71^a^	76.94 ± 12.11^a^	105.38 ± 27.25^b^	0.0386
LBP (μg/mL)	13.06 ± 4.96^a^	22.04 ± 5.33^b^	21.37 ± 5.35^b^	0.0160
Hp (μg/mL)	373.86 ± 125.63^a^	489.60 ± 209.89^a^	701.49 ± 133.22^b^	0.0098

^A-B^Means within a row differ (*P* < 0.01)

^a-b^Means within a row differ (*P* < 0.05)

^1^LA is the treatment diet based on corn grain steeped for 48 h in an equal quantity of tap water containing 1% lactic acid (wt/vol), HA is the treatment diet based on corn grain steeped for 48 h in an equal quantity of tap water containing 1% hydrochloric acid (wt/vol), and CON is the control diet containing corn grain steeped for 48 h in an equal quantity of tap water

^2^*CRP* C-reactive protein, *SAA* serum amyloid A, *LBP* lipopolysaccharide-binding protein, *Hp* haptoglobin

### Plasma cytokines concentrations

The plasma cytokines concentrations of the different diets are reported in Table 5. No differences in plasma IL-6 concentrations among the three diets were observed (*P* > 0.05), but the concentrations of plasma IL-1β and IL-8 in the LA and HA groups were lower than those in the CON group (*P* < 0.05). The plasma TNF-α concentration in the LA group was the lowest among the experimental diets (*P* < 0.05). Although the plasma TNF-α concentration in the HA group was numerically lower than that in the CON group, the difference did not attain a significant level (*P* > 0.05).

**Table 5:** The cytokines concentrations in plasma of beef steers fed different diets

Items	Diet^1^			*P*-value
	LA	HA	CON	
IL-1β^2^ (ng/L)	95.99 ± 7.33^a^	96.03 ± 9.5^a^	109.25 ± 9.36^b^	0.0287
IL-6 (ng/L)	10.19 ± 2.66	9.85 ± 3.69	10.68 ± 1.77	0.8777
IL-8 (ng/L)	110.67 ± 20.15^A^	109.94 ± 30.99^A^	163.87 ± 37.30^B^	0.0108
TNF-α (ng/L)	146.30 ± 40.35^a^	176.88 ± 23.57^ab^	198.85 ± 24.15^b^	0.0289

^A-B^Means within a row differ (*P* < 0.01)

^a-b^Means within a row differ (*P* < 0.05)

^1^LA is the treatment diet based on corn grain steeped for 48 h in an equal quantity of tap water containing 1% lactic acid (wt/vol), HA is the treatment diet based on corn grain steeped for 48 h in an equal quantity of tap water containing 1% hydrochloric acid (wt/vol), and CON is the control diet containing corn grain steeped for 48 h in an equal quantity of tap water

^2^*IL-1β* interleukin-1β, *IL-6* interleukin-6, *IL-8* interleukin-8, *TNF-α* tumour necrosis factor-alpha

### Multivariate analysis of rumen fluid and plasma metabolites

**Fig. 1:**
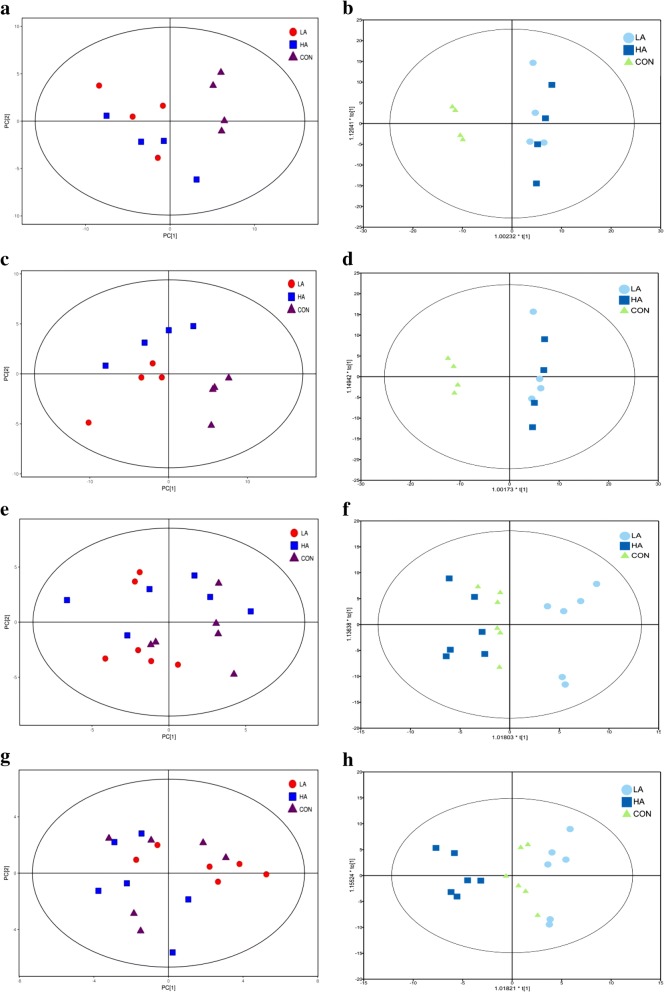
PCA score plots (**a**, **c**, **e**, and **g**) and OPLS-DA score plots (**b**, **d**, **f** and **h**) of rumen and plasma samples obtained from beef steers fed the 3 different diets. On the score plot, each point represents an individual sample. **a** and **b** are derived from the POS of the rumen fluid, and **c** and **d** are derived from the NEG of the rumen fluid. **e** and **f** are derived from the POS of plasma, and **g** and **h** are derived from the NEG of plasma. LA (circle) is the treatment diet based on corn grain steeped for 48 h in an equal quantity of tap water containing 1% lactic acid (wt/vol), HA (square) is the treatment diet based on corn grain steeped for 48 h in an equal quantity of tap water containing 1% hydrochloric acid (wt/vol), and CON (triangle) is the control diet containing corn grain steeped for 48 h in an equal quantity of tap water

The ionization source of LC-QTOFMS is ESI, including positive and negative ion modes (POS; NEG). Multivariate statistical analyses were used to explore metabolic differences among the CON, LA and HA groups. PCA, which is an unsupervised pattern recognition method, was performed to examine the intrinsic variation in metabolic patterns among the three groups. Under the supervised OPLS-DA, variable information can be extracted for the classification of large amounts of samples and for reducing unwanted systematic noise. PCA and OPLS-DA analyses of UHPLC-QTOF/MS metabolic profiles of the rumen fluid samples are illustrated in Fig. 1a, b, c and d. For ruminal samples, the distributions of the LA and HA groups overlapped and could not be well separated from each other, but the responses of beef cattle fed the CON diets were further apart from those corresponding to the LA and HA diets (Fig. 1a, b, c and d). The PCA score plot of plasma samples in the three groups could not clearly differentiate (Fig. 1e and g); however, they could be well discriminated using the OPLS-DA model (Fig. 1f and h), especially between the LA group and the other two groups.

### Significantly different metabolites among the three diets

**Fig. 2:**
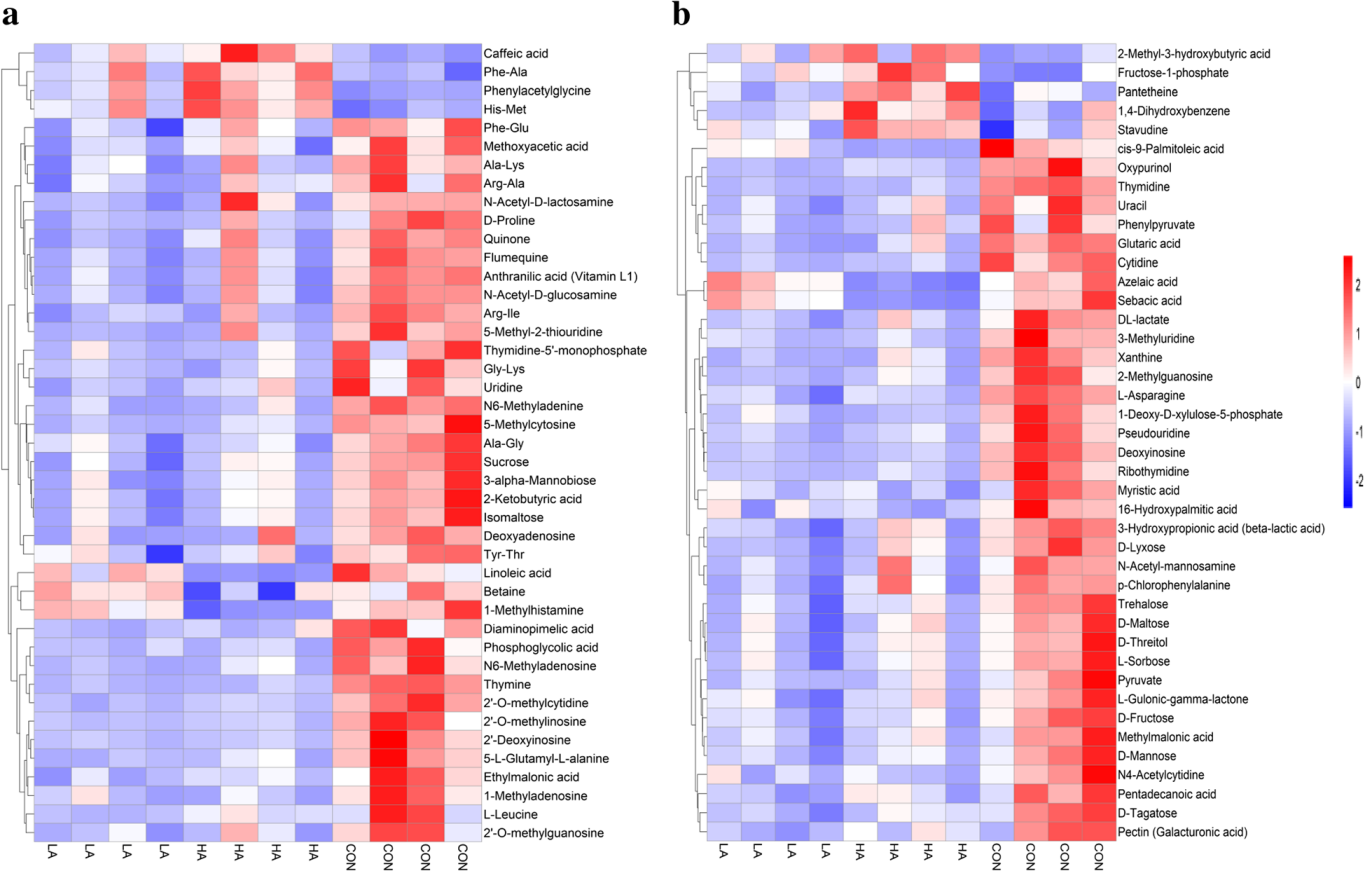
Hierarchical clustering analysis of different rumen fluid metabolites in beef cattle fed with 3 different diets. **a** and **b** are derived from the POS and NEG of the rumen fluid, respectively. Each row represents one metabolite, and each column represents one sample. Cells are coloured based on the signal intensity measured in the rumen. Red represents high rumen levels, blue shows low signal intensity, and white cells show intermediate levels. LA is the treatment diet based on corn grain steeped for 48 h in an equal quantity of tap water containing 1% lactic acid (wt/vol), HA is the treatment diet based on corn grain steeped for 48 h in an equal quantity of tap water containing 1% hydrochloric acid (wt/vol), and CON is the control diet containing corn grain steeped for 48 h in an equal quantity of tap water

As shown in Fig. 2a and b, a total of 85 rumen metabolites (POS: 43 and NEG: 42) with statistical significance (*P* < 0.05) and VIP > 1 were identified as major differentiating substances among the 3 groups. Most rumen metabolites were markedly increased by the CON diet relative to the LA or HA diet. First, all carbohydrate metabolites were found at the highest relative abundance in the beef steers fed the CON diet; these carbohydrate metabolites included 3-alpha-mannobiose, D-fructose, D-lyxose, D-maltose, D-mannose, D-tagatose, D-threitol, isomaltose, L-sorbose, sucrose, trehalose, pyruvate and so forth. The relative abundance of these carbohydrate metabolites was the lowest in the LA group. Moreover, amino acid metabolites of beef cattle fed the CON diet were also found at the highest relative abundance; these metabolites included Ala-Gly, Ala-Lys, Arg-Ala, Arg-Ile, D-proline, Gly-Lys, L-asparagine, L-leucine, Phe-Glu and Tyr-Thr, except for His-Met and Phe-Ala. With regard to nucleotide metabolites, the relative abundances (%) of thymine, uracil and xanthine in the CON group were more than 11.84-fold and 8.56-fold, 2.57-fold and 1.89-fold, 3.40-fold and 2.48-fold higher than those in the LA and HA groups, respectively (Additional file 1: Table S1). Finally, the relative abundances of DL-lactate, beta-lactic acid and glutaric acid were the highest in the CON group, followed by the HA group, and the lowest in the LA group.

**Fig. 3:**
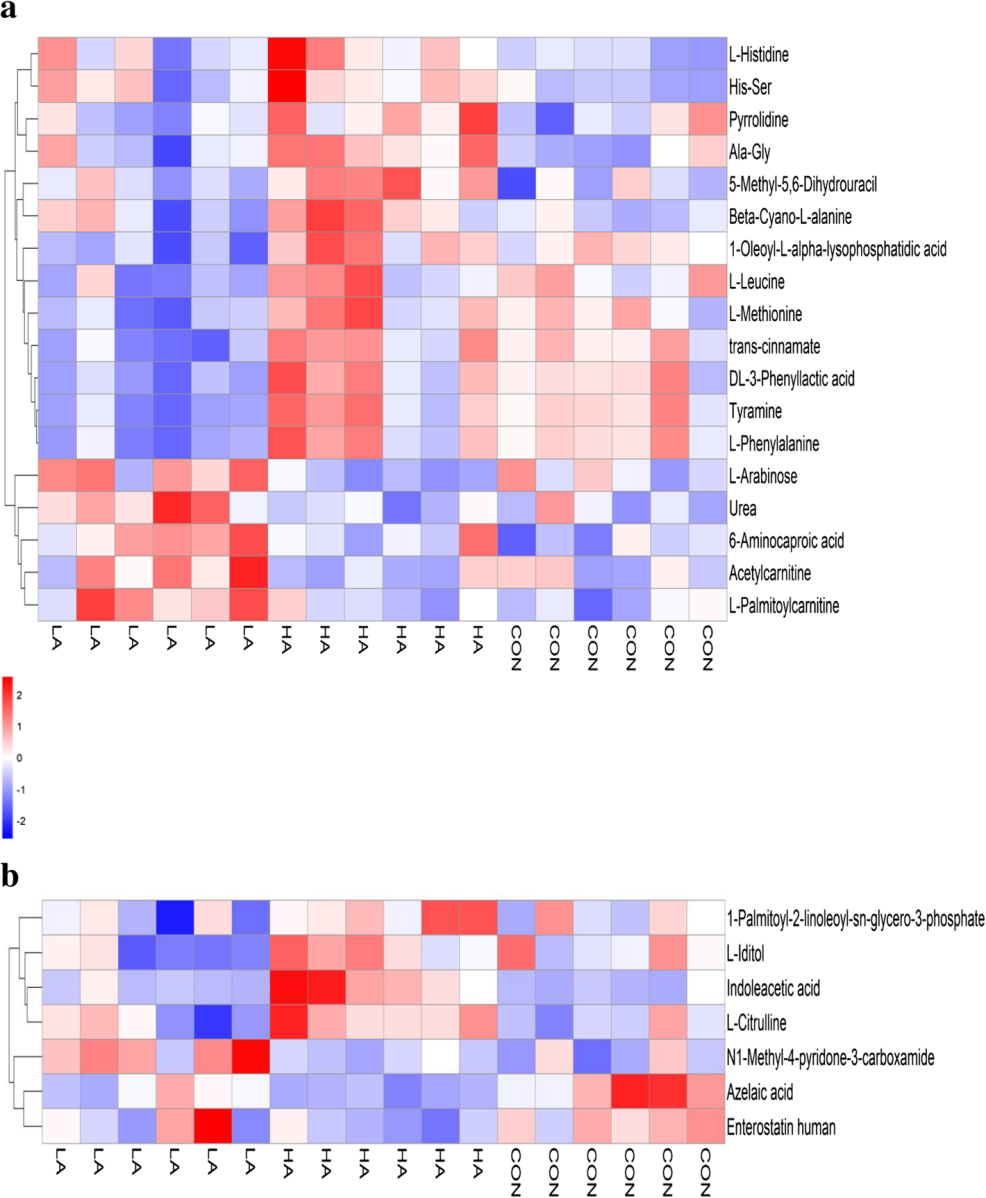
Hierarchical clustering analysis of different plasma metabolites in beef cattle fed with 3 different diets. **a** and **b** are derived from the POS and NEG of the plasma, respectively. Each row represents one metabolite, and each column represents one sample. Cells are coloured based on the signal intensity measured in plasma. Red represents high plasma levels, blue shows low signal intensity, and white cells show intermediate levels. LA is the treatment diet based on corn grain steeped for 48 h in an equal quantity of tap water containing 1% lactic acid (wt/vol), HA is the treatment diet based on corn grain steeped for 48 h in an equal quantity of tap water containing 1% hydrochloric acid (wt/vol), and CON is the control diet containing corn grain steeped for 48 h in an equal quantity of tap water

Figure 3 lists 25 plasma different metabolites from the three groups. Several compounds in the plasma have attracted attention as significantly different metabolites. For example, the relative abundance of tyramine and DL-3-phenyllactic acid in the LA group was the lowest compared with those in the other groups (Additional file 2: Table S2). L-citrulline, L-leucine, L-methionine, and L-phenylalanine, which are involved in amino acid metabolism, were also the lowest in the LA group, but the related histidine metabolites, such as His-Ser and L-Histidine, were the lowest in the CON group, followed by the LA group, and the highest in the HA group (Additional file 2: Table S2). The relative abundance of above amino acids in the plasma was the highest in the HA group, and the relative abundance of urea in plasma was the highest in the LA group and the lowest in HA group (Additional file 2: Table S2).

## Discussion

Although corn steeped in 1% LA or 1% HA had higher acidity (Additional file 3: Table S3), the results showed that there was no significant difference in ADMI among the three treatments. Mickdam et al. [25] also reported that diets including barley treated with 0.5% or 1% LA did not exert a negative effect on DMI in early-lactating cows.

Feeding a high-grain diet can result in the accumulation of organic acids in the rumen and the weakening of rumen buffering, which can lead to a decrease in the rumen pH [26, 27]. Our results demonstrated that steeping corn grain in 1% LA or 1% HA significantly increased the rumen pH. The greater rumen pH was attributed to the augmentation of the ruminal resistant starch (RRS) for lowering concentrations of VFA in the rumen. Indeed, our results showed that the LA or HA treatment reduced the concentrations of acetate, iso-butyrate and iso-valerate, although propionate, butyrate and valerate were not significantly different in this study. In addition, LA and HA treatments may increase the activity of lactate-utilizing bacteria for reducing the concentration of lactate in rumen. Compared with VFA normally associated with ruminal fermentation, the p*K*_a_ of lactate is lower (3.9 for lactate versus 4.9 for VFA) [28], and reduction of ruminal lactate production was more conducive to increasing rumen pH values. Mao et al. [28] indicated that disodium fumarate did not affect the production of total VFA or its individual acids, and ruminal pH increased linearly as the amount of disodium fumarate added increased, while lactate production decreased linearly. The results of rumen metabolomics in the present study showed that the relative abundance of DL-lactate, as a differential metabolite among the three diets, was decreased in LA and HA groups compared with the CON group. Thus, the higher ruminal pH of beef cattle fed corn grain steeped in 1% LA or 1% HA may result from the low production of VFA and lactate in the rumen.

LPS is a component of the cell wall of Gram-negative bacteria, and it has been widely recognized that ruminal LPS concentrations increase after grain engorgement. Zhou et al. [29] found that the rumen pH of cows fed a high-concentrate diet with a concentrate to roughage ratio of 65:35 and a low-concentrate diet with a concentrate to roughage ratio of 46:54 were 6.21 and 6.62, and the rumen LPS concentrations were 29,065 EU/mL and 11,664 EU/mL, respectively. A decline in ruminal pH causes death and cell lysis of gram-negative bacteria, resulting in an increase in the ruminal LPS concentration [30]. As stated above, the LA and HA treatments contributed to reducing the concentrations of organic acids in the rumen (i.e., acetate, iso-butyrate, iso-valerate and DL-lactate) and thus increased the ruminal pH. Therefore, treatment with 1% LA or 1% HA tended (*P = 0.0572*) to decrease the concentrations of LPS in the rumen. In addition, LPS released during the growth of bacteria may account for as much as 60% of that released in the rumen [4]. During rapid growth, autolytic enzymes are required to help cells expand and grow; however, excessive autolytic activity can lead to bacterial cell apoptosis and lysis. *E. coli*, a Gram-negative bacterium in the rumen, is the major contributor to the rumen LPS pool [31, 32]. There is a specific maltose-binding protein located in the periplasmic space of the cell wall of *E. coli* [33]. The finding from our study was that the relative abundance of D-maltose was the lowest in the LA group, followed by that in the HA group, and the highest in the CON group, which was consistent with the change in the ruminal LPS concentrations among the 3 groups.

LPS produced in the digestive tract can be translocated into the bloodstream; thus, the concentration of blood LPS increases [4]. As a result, immune responses are activated by circulating LPS, and the blood concentrations of acute phase proteins, such as SAA, Hp, LBP and CRP, increase [34–36]. In the current study, compared with the CON group, the concentrations of plasma LPS in the LA group decreased significantly. Meanwhile, the plasma concentrations of SAA, CRP and Hp in the LA and HA groups were significantly lower than those in the CON group, and the plasma LBP concentrations in the LA group were also significantly lower than those in the CON group. The present findings are consistent with those of Iqbal, who found lower concentrations of plasma Hp and SAA in dairy cows fed LA-treated barley [37]. Moreover, the translocation of LPS into the systemic circulation stimulates the release of pro-inflammatory cytokines, such as IL-1, IL-6 and TNF-α, by mononuclear phagocytes [29]. Our study demonstrated that the release of cytokines (IL-1β, IL-8 and TNF-α) in plasma decreased when beef steers were fed the LA- or HA-treated corn. IL-6 is a complex cytokine that enhances or limits the immune response [29]. The concentrations of IL-6 were not different among the three groups, which may be attributed to the fact that IL-6 is a duplicitous cytokine that plays both pro- and anti-inflammatory roles. These findings indicated that steeping corn in LA or HA had beneficial effects in beef steers fed high-concentrate diets.

In addition to evaluate the effect of feeding corn steeped in LA or HA on inflammatory responses in beef steers, we also explored the evidence of LA or HA treatment on changing the metabolomic profiles in the rumen and plasma by UHPLC-QTOF-MS metabolomics analysis. Based on each PCA scot plot and OPLS-DA scot plot, the experimental results clearly showed significant differences in the ruminal metabolites among the basal diet and the two experimental diets. This study indicated that the metabolic profile changed in association with the LA- and HA-treated corn.

The main component of most cereal grains is starch, and in this present study the content of starch in corn grain steeped with 1% LA, 1% HA and water was 63.4, 66.0 and 71.8% of the dry matter (DM), respectively. Non-structural carbohydrates have fast rumen degradation as opposed to the slow degradation rate of structural carbohydrates. Non-structural carbohydrates are easily degraded in the rumen by protozoa that engulf starch granules or amylolytic bacteria that secrete α-amylases [11]. Oligosaccharides, dextrines and small amounts of free glucose are the end products of amylose and amylopectin debranching before treatment with other oligosaccharides [38]. In the current study, all qualitative metabolites of carbohydrates were found at the highest relative abundance in the CON-fed beef steers compared with the LA- or HA-fed beef steers; these metabolites included 3-Alpha-mannobiose, D-fructose, D-lyxose, D-maltose, D-mannose, D-tagatose, D-threitol, isomaltose, L-sorbose, sucrose, and trehalose. In addition, pyruvate, an intermediate product of starch degradation in the rumen [39], was approximately 2-fold higher in the CON group than in the LA group or the HA group (Additional file 1: Table S1). These results indicated that corn starch can escape microbial degradation in the rumen after being steeped in LA or HA. Östman et al. [13] observed that the hydrolysis indices (HI) of a starch and gluten mixture containing lactic acid were 22% lower than the corresponding mixture without the acid. Khol-Parisini et al. [40] also found that treatment of barley grain in 1% LA modulated the in situ rumen degradation kinetics of starch in cows. In general, the results obtained by our study further provided powerful evidence of the modulation of starch characteristics by chemical processing.

Reduced starch degradation in the rumen would allow more starch to be digested in the intestine. The change in the site of starch digestion from the rumen to the intestine could also be responsible for some of the changes seen in protein metabolism [41]. Interestingly, most amino acid metabolites of beef cattle fed the CON diet were also found at the highest relative abundance in this study; these metabolites included Ala-Gly, Ala-Lys, Arg-Ala, Arg-Ile, D-proline, Gly-Lys, L-asparagine, L-leucine, Phe-Glu and Tyr-Thr. The structural elements of grains are the pericarp, the inner germ (embryo) and endosperm. The endosperm contains the major part of starch and consists of four layers, starting with the outer aleurone layer, followed by the subaleurone layer (peripheral endosperm), corneous endosperm, and the inner floury endosperm. The peripheral endosperm and corneous endosperm embody starch granules and are enclosed by an impenetrable protein matrix [11]. Because of the structural cross-linking changes in the protein matrix, proteolysis of the protein may be reduced and in turn reduce the degradation of the starch surrounded by the protein matrix. Iqbal [42] suggested that the composition and the ability of the protein matrix to be accessed by microorganisms are major control points for the rate of starch degradation. Our results also indicated that the concentrations of iso-butyrate and iso-valerate in rumen fluid of beef cattle in the LA group and the HA group were significantly lower than those in the CON group. This was in agreement with the study by Deckardt et al. [43] that reported that iso-butyrate and iso-valerate decreased in response to LA treatment. All amino acids that are not linked or guarded from attack are extensively degraded in the rumen to ammonia, carbon dioxide, VFA, and branch-chain fatty acids [44]. Therefore, the decrease in branch-chain fatty acids indicated reduced degradation of amino acids from dietary protein.

A stable and healthy population of ruminal microbiota is critical for maintaining the health of ruminants. Saleem and colleagues conducted several metabolic experiments to reveal alterations in rumen metabolism with an increased proportion of cereal grain in the diet of dairy cows [33, 45, 46]. All their results showed that the rumen concentrations of urea, hypoxanthine, xanthine, uracil and LPS were elevated with increasing proportions of grain. The above metabolites are degradation products of rumen bacteria. These findings indicated that a high-grain diet may disturb the microbial community. Mao et al. [30] also confirmed that high maize feeding has a significantly negative impact on the ruminal biodiverse ecosystem. An interesting observation arising from our study was that the relative abundance of uracil and xanthine in the LA or HA groups was lower than that of the CON group, which may alleviate the negative effects of high-cereal diets on rumen microorganisms.

Regarding the results of the plasma differential metabolites, the most interesting finding was related to the phenylalanine metabolism pathway. Our previous experiments showed that phenylalanine metabolism was the common key metabolic pathway involved in a high-grain diet (unpublished). L-phenylalanine and many metabolites involved in this pathway were significantly increased with the high-grain diet; for example, there was higher plasma content of phenyllactic acid and tyramine (unpublished). Our current results further confirmed the role of this metabolic pathway in the metabolism of high-grain diets. Compared with the CON group, the relative abundance of L-phenylalanine, DL-3-phenyllactic acid and tyramine significantly decreased in the LA group. The main metabolic pathway of phenylalanine is to produce tyrosine by the catalysis of phenylalanine hydroxylase. Tyramine is produced in the metabolic process of tyrosine. Tyramine is a kind of biogenic amine; low concentrations of biogenetic amines are essential for the normal growth and differentiation of cells, but higher concentrations can delay epithelial regeneration and induce epithelial damage [30]. Our findings indicated that LA treatment can improve the negative effects of a high-grain diet, which is beneficial to the health of beef cattle. In contrast to the LA group, these relative abundances in the HA group increased. The specific mechanism explaining the incremental effect of HA treatment on L-phenylalanine, DL-3-phenyllactic acid and tyramine in this research is not very well understood at present. In addition, the relative abundance of His-Ser and L-Histidine in plasma was the lowest in the CON group compared with that in the LA and HA groups. Histidine metabolism is related to another biogenic amine (i.e., histamine). Histamine in the rumen is derived from the amino carboxylic acid of histidine via a decarboxylation reaction [3]. The relative abundance (%) of ruminal histamine in the three groups was 23.04 (LA), 31.15 (HA) and 45.81 (CON) (*P = 0.18*, VIP = 1.39). The pH value of the gastrointestinal tract is a crucial factor affecting the activity of microbial amino acid decarboxylase. In fact, it was reported that the activity of the amino acid decarboxylase was greater when pH decreased [47]. The results of the present study indicated that the LA or HA treatment increased the rumen pH and inhibited the decarboxylation of histidine in the rumen, which helped more histidine to be absorbed.

When nitrogen availability in the rumen is relatively high when compared to carbohydrate availability, a great amount of ammonia is produced inside rumen, and the main nitrogen flow goes from the rumen to the bloodstream. In this case, there will be great concentrations of blood urea [44]. In this study, the relative abundance of plasma urea in the LA treatment was higher, which indicated that the degradation of grain after LA treatment in the rumen decreased. However, the increase in the concentration of urea in the blood causes losses of nitrogen. The disadvantage effect of LA treatment needs further experimental confirmation. However, the relative abundance of urea in plasma was the lowest in the HA group, which indicated that most of urea is excreted to the rumen so that it can be utilized in the synthesis of proteins that will contribute to the host’s amino acid needs, and in this study the relative abundance of all amino acids in the plasma was the highest in the HA group.

## Conclusions

Overall, based on UHPLC-QTOF/MS metabolomics and multivariate analyses, this study showed that steeping corn in 1% LA or 1% HA modulated the metabolic profiles of the rumen. Feeding beef steers corn steeped in 1% LA or 1% HA was associated with lower relative abundance of carbohydrate metabolites, amino acid metabolites, xanthine, uracil, DL-lactate, acetate, iso-butyrate and iso-valerate in the rumen as well as with higher ruminal pH and a tendency for lower ruminal LPS concentrations. Moreover, the data showed lower concentrations of plasma CRP, SAA, Hp, IL-1β, and IL-8 in beef steers fed diets containing corn treated with 1% LA or 1% HA. The 1% LA treatment decreased the concentrations of plasma LPS, LBP and TNF-α and the relative abundance of L-phenylalanine, DL-3-phenyllactic acid and tyramine in plasma. The 1% HA treatment decreased the relative abundance of urea in plasma and increased the relative abundance of all amino acids in plasma. These findings indicated that LA or HA treatment of corn modulated the degradation characteristics of starch, which contributed to improving the rumen and plasma metabolic profiles and to decreasing inflammatory responses in beef steers fed a high-concentrate diet.

## Additional files

Additional file 1: **Table S1.** Relative distributions (%) of the different ruminal metabolites among the three groups. (DOCX 45 kb)

Additional file 2: **Table S2.** Relative distributions (%) of the different plasma metabolites among the three groups. (DOCX 18 kb)

Additional file 3: **Table S3.** pH values of corn and total mixed ration in three treatments. (DOCX 16 kb)

## Abbreviations

ADMI: Average daily dry matter intake
CE: Collision energy
CON: Control diet
CRP: C-reactive protein
DM: Dry matter
DMI: Dry matter intake
ESI: Electron spray ionization
HA: Hydrochloric acid
HI: Hydrolysis indices
Hp: Haptoglobin
IDA: Information-dependent basis
IL-1β: Interleukin-1β
IL-6: Interleukin-6
IL-8: Interleukin-8
ISVF: Ion spray voltage floating
LA: Lactic acid
LBP: Lipopolysaccharide-binding protein
LPS: Lipopolysaccharide
NEG: Negative ion mode
OPLS-DA: Orthogonal partial least-squares discriminant analysis
PCA: Principal component analysis
POS: Positive ion mode
RRS: Ruminal resistant starch
RT: Retention time
SAA: Serum amyloid A
SARA: Subacute ruminal acidosis
TMR: Total mixed ration
TNF-α: Tumour necrosis factor-alpha
UHPLC-QTOF/MS: Ultra-high-performance liquid tandem chromatography-quadrupole time-of-flight mass spectrometry
VFA: Volatile fatty acid
VIP: Variable importance for the projection
