# Supplementary material for: Treatment of corn with lactic acid or hydrochloric acid modulates the rumen and plasma metabolic profiles as well as inflammatory responses in beef steers
Source: BMC Vet Res. 2018 Dec 18;14:408. doi: 10.1186/s12917-018-1734-3 (PMC6299609; doi:10.1186/s12917-018-1734-3)
Supplement: Supplementary file 1 — Table S1. Relative distributions (%) of the different ruminal metabolites among the three groups. (DOCX 45 kb) [file 12917_2018_1734_MOESM1_ESM.docx]

Table S1 Relative distributions (%) of the different ruminal metabolites among the three groups

| Name | Relative abundance (%) | | | *P*-value | VIP^2^ |
| --- | --- | --- | --- | --- | --- |
|  | LA^1^ | HA | CON |  |  |
| 1,4-Dihydroxybenzene | 26.35 | 50.36 | 23.29 | 0.0481 | 1.33 |
| 16-Hydroxypalmitic acid | 29.39 | 24.82 | 45.79 | 0.0247 | 1.20 |
| 1-Deoxy-D-xylulose-5-phosphate | 20.50 | 14.91 | 64.60 | 0.0295 | 1.99 |
| 1-Methyladenosine | 24.50 | 20.50 | 55.00 | 0.0144 | 1.93 |
| 1-Methylhistamine | 38.10 | 19.05 | 42.86 | 0.0013 | 1.59 |
| 2'-Deoxyinosine | 7.38 | 8.93 | 83.68 | 0.0439 | 2.08 |
| 2-Ketobutyric acid | 20.23 | 26.11 | 53.66 | 0.0110 | 1.59 |
| 2-Methyl-3-hydroxybutyric acid | 33.33 | 48.33 | 18.33 | 0.0412 | 1.55 |
| 2-Methylguanosine | 15.29 | 22.35 | 62.35 | 0.0401 | 1.91 |
| 2'-O-methylcytidine | 15.09 | 18.87 | 66.04 | 0.0055 | 2.14 |
| 2'-O-methylguanosine | 19.19 | 26.26 | 54.55 | 0.0372 | 1.58 |
| 2'-O-methylinosine | 15.38 | 18.46 | 66.15 | 0.0410 | 2.15 |
| 3-alpha-Mannobiose | 17.82 | 26.05 | 56.13 | 0.0058 | 1.61 |
| 3-Hydroxypropionic acid (beta-lactic acid) | 20.81 | 29.44 | 49.75 | 0.0055 | 1.57 |
| 3-Methyluridine | 14.76 | 14.76 | 70.48 | 0.0040 | 1.96 |
| 5-L-Glutamyl-L-alanine | 18.97 | 24.14 | 56.90 | 0.0373 | 2.02 |
| 5-Methyl-2-thiouridine | 9.83 | 26.92 | 63.24 | 0.0210 | 1.71 |
| 5-Methylcytosine | 12.06 | 18.44 | 69.50 | 0.0006 | 2.02 |
| Ala-Gly | 26.80 | 26.80 | 46.39 | 0.0036 | 1.87 |
| Ala-Lys | 24.19 | 29.03 | 46.77 | 0.0381 | 1.46 |
| Anthranilic acid (Vitamin L1) | 22.03 | 28.99 | 48.99 | 0.0106 | 1.74 |
| Arg-Ala | 26.04 | 31.25 | 42.71 | 0.0341 | 1.40 |
| Arg-Ile | 26.47 | 29.41 | 44.12 | 0.0040 | 1.91 |
| Azelaic acid | 41.87 | 13.45 | 44.67 | 0.0009 | 1.29 |
| Betaine | 37.06 | 26.28 | 36.66 | 0.0325 | 1.00 |
| Caffeic acid | 30.89 | 57.46 | 11.66 | 0.0097 | 1.94 |
| cis-9-Palmitoleic acid | 31.27 | 11.35 | 57.38 | 0.0194 | 1.74 |
| Cytidine | 14.55 | 16.36 | 69.09 | 0.0002 | 2.10 |
| Deoxyadenosine | 16.81 | 26.74 | 56.45 | 0.0498 | 1.62 |
| Deoxyinosine | 7.47 | 9.24 | 83.29 | 0.0089 | 2.04 |
| D-Fructose | 20.66 | 24.41 | 54.93 | 0.0053 | 1.62 |
| Diaminopimelic acid | 25.76 | 28.79 | 45.45 | 0.0380 | 1.79 |
| DL-lactate | 16.79 | 25.98 | 57.22 | 0.0081 | 1.65 |
| D-Lyxose | 15.90 | 27.13 | 56.97 | 0.0038 | 1.62 |
| D-Maltose | 21.58 | 29.28 | 49.14 | 0.0225 | 1.39 |
| D-Mannose | 18.57 | 25.14 | 56.29 | 0.0094 | 1.54 |
| D-Proline | 20.97 | 29.03 | 50.00 | 0.0128 | 1.70 |
| D-Tagatose | 17.68 | 27.23 | 55.10 | 0.0061 | 1.50 |
| D-Threitol | 22.34 | 29.92 | 47.75 | 0.0234 | 1.41 |
| Ethylmalonic acid | 19.05 | 26.19 | 54.76 | 0.0103 | 1.77 |
| Flumequine | 16.42 | 27.81 | 55.77 | 0.0141 | 1.60 |
| Fructose-1-phosphate | 32.93 | 46.95 | 20.12 | 0.0114 | 1.79 |
| Glutaric acid | 14.56 | 23.42 | 62.03 | 0.0003 | 1.92 |
| Gly-Lys | 19.10 | 19.10 | 61.80 | 0.0073 | 1.94 |
| His-Met | 33.33 | 41.43 | 25.24 | 0.0047 | 1.98 |
| Isomaltose | 19.31 | 25.62 | 55.07 | 0.0085 | 1.58 |
| L-Asparagine | 23.26 | 27.91 | 48.84 | 0.0001 | 1.91 |
| L-Gulonic-gamma-lactone | 23.86 | 28.55 | 47.59 | 0.0249 | 1.50 |
| Linoleic acid | 41.27 | 6.88 | 51.85 | 0.0112 | 1.53 |
| L-Leucine | 15.67 | 29.40 | 54.94 | 0.0418 | 1.34 |
| L-Sorbose | 21.93 | 28.31 | 49.76 | 0.0167 | 1.46 |
| Methoxyacetic acid | 24.22 | 29.69 | 46.09 | 0.0281 | 1.54 |
| Methylmalonic acid | 21.84 | 27.62 | 50.54 | 0.0270 | 1.43 |
| Myristic acid | 30.29 | 27.49 | 42.22 | 0.0190 | 1.71 |
| N4-Acetylcytidine | 28.72 | 27.66 | 43.62 | 0.0223 | 1.64 |
| N6-Methyladenine | 14.54 | 20.21 | 65.25 | 0.0001 | 1.98 |
| N6-Methyladenosine | 11.17 | 19.68 | 69.15 | 0.0267 | 2.59 |
| N-Acetyl-D-glucosamine | 20.66 | 28.17 | 51.17 | 0.0071 | 1.74 |
| N-Acetyl-D-lactosamine | 22.73 | 36.36 | 40.91 | 0.0020 | 1.34 |
| N-Acetyl-mannosamine | 19.74 | 30.70 | 49.56 | 0.0462 | 1.46 |
| Oxypurinol | 18.50 | 23.35 | 58.15 | 0.0145 | 2.11 |
| Pantetheine | 23.38 | 51.95 | 24.68 | 0.0027 | 1.13 |
| p-Chlorophenylalanine | 20.00 | 33.33 | 46.67 | 0.0295 | 1.38 |
| Pectin (Galacturonic acid) | 16.61 | 29.15 | 54.24 | 0.0253 | 1.30 |
| Pentadecanoic acid | 21.68 | 29.45 | 48.87 | 0.0162 | 1.55 |
| Phe-Ala | 32.88 | 43.84 | 23.29 | 0.0168 | 1.84 |
| Phe-Glu | 28.33 | 33.33 | 38.33 | 0.0122 | 1.38 |
| Phenylacetylglycine | 32.42 | 43.53 | 24.05 | 0.0046 | 1.97 |
| Phenylpyruvate | 24.32 | 29.69 | 46.00 | 0.0380 | 1.63 |
| Phosphoglycolic acid | 16.98 | 16.98 | 66.04 | 0.0303 | 2.18 |
| Pseudouridine | 15.73 | 19.67 | 64.60 | 0.0039 | 1.87 |
| Pyruvate | 22.65 | 25.78 | 51.57 | 0.0231 | 1.76 |
| Quinone | 23.68 | 31.58 | 44.74 | 0.0066 | 1.65 |
| Ribothymidine | 11.32 | 13.96 | 74.72 | 0.0354 | 2.05 |
| Sebacic acid | 40.83 | 9.37 | 49.80 | 0.0058 | 1.37 |
| Stavudine | 31.50 | 39.31 | 29.19 | 0.0394 | 1.36 |
| Sucrose | 20.18 | 28.07 | 51.75 | 0.0037 | 1.59 |
| Thymidine | 7.66 | 10.47 | 81.87 | 0.0000 | 2.13 |
| Thymidine-5'-monophosphate | 18.75 | 18.75 | 62.50 | 0.0237 | 1.71 |
| Thymine | 7.03 | 9.72 | 83.25 | 0.0000 | 2.14 |
| Trehalose | 22.76 | 28.97 | 48.28 | 0.0044 | 1.62 |
| Tyr-Thr | 27.45 | 29.41 | 43.14 | 0.0492 | 1.40 |
| Uracil | 20.30 | 27.62 | 52.09 | 0.0075 | 1.75 |
| Uridine | 17.19 | 28.13 | 54.69 | 0.0168 | 1.67 |
| Xanthine | 17.31 | 23.78 | 58.92 | 0.0009 | 1.99 |

^1^LA is the treatment diet based on corn grain steeped for 48 h in an equal quantity of tap water containing 1% lactic acid (wt/vol), HA is the treatment diet based on corn grain steeped for 48 h in an equal quantity of tap water containing 1% hydrochloric acid (wt/vol), and CON is the control diet containing corn grain steeped for 48 h in an equal quantity of tap water.

^2^Variable importance in the projection (VIP) was obtained from OPLS-DA model with value higher than 1.0.
