# Supplementary material for: Treatment of corn with lactic acid or hydrochloric acid modulates the rumen and plasma metabolic profiles as well as inflammatory responses in beef steers
Source: BMC Vet Res. 2018 Dec 18;14:408. doi: 10.1186/s12917-018-1734-3 (PMC6299609; doi:10.1186/s12917-018-1734-3)
Supplement: Supplementary file 2 — Table S2. Relative distributions (%) of the different plasma metabolites among the three groups. (DOCX 18 kb) [file 12917_2018_1734_MOESM2_ESM.docx]

Table S2 Relative distributions (%) of the different plasma metabolites among the three groups

| Name | Relative abundance (%) | | | *P*-value | VIP^2^ |
| --- | --- | --- | --- | --- | --- |
|  | LA^1^ | HA | CON |  |  |
| Beta-Cyano-L-alanine | 29.79 | 40.43 | 29.79 | 0.0289 | 1.64 |
| 1-Oleoyl-L-alpha-lysophosphatidic acid | 22.97 | 41.72 | 35.32 | 0.0005 | 2.46 |
| 1-Palmitoyl-2-linoleoyl-sn-glycero-3-phosphate | 29.89 | 36.90 | 33.21 | 0.0477 | 2.13 |
| 5-Methyl-5,6-dihydrouracil | 30.34 | 40.80 | 28.87 | 0.0046 | 1.51 |
| 6-Aminocaproic acid | 36.66 | 32.97 | 30.37 | 0.0127 | 1.19 |
| Acetylcarnitine | 42.23 | 26.93 | 30.84 | 0.0405 | 1.59 |
| Ala-Gly | 31.11 | 37.78 | 31.11 | 0.0078 | 1.75 |
| Azelaic acid | 32.23 | 22.31 | 45.45 | 0.0046 | 1.56 |
| DL-3-Phenyllactic acid | 27.66 | 38.30 | 34.04 | 0.0033 | 2.19 |
| Enterostatin human | 34.78 | 27.54 | 37.68 | 0.0174 | 1.14 |
| His-Ser | 32.32 | 40.85 | 26.83 | 0.0355 | 1.10 |
| Indoleacetic acid | 20.79 | 61.39 | 17.82 | 0.0108 | 2.19 |
| L-Arabinose | 46.15 | 20.51 | 33.33 | 0.0063 | 2.13 |
| L-Citrulline | 30.04 | 39.09 | 30.86 | 0.0238 | 1.85 |
| L-Histidine | 31.75 | 41.25 | 27.00 | 0.0296 | 1.12 |
| L-Iditol | 28.57 | 36.61 | 34.82 | 0.0171 | 1.62 |
| L-Leucine | 28.30 | 36.38 | 35.32 | 0.0247 | 1.93 |
| L-Methionine | 26.97 | 38.20 | 34.83 | 0.0066 | 2.09 |
| L-Palmitoylcarnitine | 43.59 | 29.49 | 26.92 | 0.0059 | 1.33 |
| L-Phenylalanine | 27.12 | 37.26 | 35.62 | 0.0018 | 2.21 |
| N1-Methyl-4-pyridone-3-carboxamide | 45.45 | 27.27 | 27.27 | 0.0042 | 1.31 |
| Pyrrolidine | 29.61 | 39.47 | 30.92 | 0.0460 | 1.57 |
| Trans-cinnamate | 27.91 | 37.21 | 34.88 | 0.0008 | 2.26 |
| Tyramine | 26.76 | 37.43 | 35.81 | 0.0017 | 2.20 |
| Urea | 37.68 | 30.77 | 31.55 | 0.0123 | 1.66 |

^1^LA is the treatment diet based on corn grain steeped for 48 h in an equal quantity of tap water containing 1% lactic acid (wt/vol), HA is the treatment diet based on corn grain steeped for 48 h in an equal quantity of tap water containing 1% hydrochloric acid (wt/vol), and CON is the control diet containing corn grain steeped for 48 h in an equal quantity of tap water.

^2^Variable importance in the projection (VIP) was obtained from OPLS-DA model with value higher than 1.0.
