# Supplementary material for: Treatment of corn with lactic acid or hydrochloric acid modulates the rumen and plasma metabolic profiles as well as inflammatory responses in beef steers
Source: BMC Vet Res. 2018 Dec 18;14:408. doi: 10.1186/s12917-018-1734-3 (PMC6299609; doi:10.1186/s12917-018-1734-3)
Supplement: Supplementary file 3 — Table S3. pH values of corn and total mixed ration in three treatments. (DOCX 16 kb) [file 12917_2018_1734_MOESM3_ESM.docx]

Table S3 pH values of corn and total mixed ration in three treatments

| Items | Diet^1^ | | |
| --- | --- | --- | --- |
|  | LA | HA | CON |
| Corn grain | 1.0 | 4.0 | 6.0 |
| Total mixed ration | 3.0 | 5.0 | 6.0 |

^1^LA is the treatment diet based on corn grain steeped for 48 h in an equal quantity of tap water containing 1% lactic acid (wt/vol), HA is the treatment diet based on corn grain steeped for 48 h in an equal quantity of tap water containing 1% hydrochloric acid (wt/vol), and CON is the control diet containing corn grain steeped for 48 h in an equal quantity of tap water.
